# The Stability study: a protocol for a multicenter randomized clinical trial comparing anterior cruciate ligament reconstruction with and without Lateral Extra-articular Tenodesis in individuals who are at high risk of graft failure

**DOI:** 10.1186/s12891-019-2589-x

**Published:** 2019-05-15

**Authors:** Alan Getgood, Dianne Bryant, Andrew Firth, Stability Group, Alan Getgood, Robert Litchfield, Kevin Willits, Chris Hewison, Nicole Kaniki, Alliya Remtulla, Stacey Wanlin, Andrew Firth, Ryan Pinto, Ashley Martindale, Lindsey O’Neill, Morgan Jennings, Michal Daniluk, Dianne Bryant, Trevor Birmingham, Dory Boyer, Bob McCormack, Mauri Zomar, Karyn Moon, Raely Moon, Brenda Fan, Bindu Mohan, Mark Heard, Greg Buchko, Laurie Hiemstra, Sarah Kerslake, Meagan Heard, Peter MacDonald, Greg Stranges, Sheila Mcrae, Lee Anne Gullett, Holly Brown, Alexandra Legary, Alison Longo, Mat Christian, Celeste Ferguson, Alex Rezansoff, Nick Mohtadi, Rhamona Barber, Denise Chan, Caitlin Campbell, Alexandra Garven, Karen Pulsifer, Michelle Mayer, Devin Peterson, Nicole Simunovic, Andrew Duong, Zakia Islam, Davide Bardana, Fiona Howells, Murray Tough, Tim Spalding, Pete Thompson, Andrew Metcalfe, Juul Achten, Michael Thomas, Andrew Legg, Lior Laver, Laura Asplin, Alisen Dube, Louise Clarkson, Jaclyn Brown, Alison Bolsover, Carolyn Bradshaw, Larissa Belgrove, Francis Millan, Sylvia Turner, Sarah Verdugo, Janet Lowe, Debra Dunne, Kerri McGowan, Charlie-Marie Suddens, Peter Verdonk, Geert Declerq, Kristien Vuylsteke, Mieke Van Haver

**Affiliations:** 1 Orthopaedic Sport Medicine Fellowship Director, Fowler Kennedy Sport Medicine Clinic, 3M Centre, University of Western Ontario, 1151 Richmond St., London, ON N6A 3K7 Canada; 2 Faculty of Health Sciences, Elborn College, University of Western Ontario, Room 1438, 1201 Western Rd, London, ON N6C 1H1 Canada; 3 Fowler Kennedy Sport Medicine Clinic, 3M Centre, University of Western Ontario, 1151 Richmond St, London, ON N6A 3K7 Canada

**Keywords:** Orthopaedics, Anterior cruciate ligament (ACL) repair, Anterolateral ligament, Tenodesis

## Abstract

**Background:**

The purpose of anterior cruciate ligament reconstruction (ACLR) is to restore stability to the knee. Persistent rotational laxity following ACLR has been correlated with poor outcome and graft failure. We hypothesize that anterolateral complex reconstruction by way of a Modified Lemaire Lateral Extra-articular Tenodesis (LET) in combination with single bundle ACLR would reduce the risk of persistent rotatory laxity in young individuals who are deemed as being at high risk of failure. We will conduct a pragmatic, multicenter, randomized clinical trial comparing standard single bundle hamstring ACLR with combined ACLR and LET.

**Methods:**

Six-hundred patients (300 per group) aged 25 years or less with an ACL deficient knee that meet two of the following three criteria will be included: 1) Grade 2 pivot shift or greater; 2) Returning to high risk cutting or pivoting sports; 3) Generalized ligamentous laxity. Participants will be seen at 3-months, 6-months, 12-months and 24-months post-operatively. The primary outcome measure is graft failure requiring revision ACLR or symptomatic instability associated with a positive asymmetric pivot shift indicating persistent rotational laxity. Patients will complete secondary outcome measures at each follow-up visit including patient-reported outcome measures, functional and biomechanical testing, and magnetic resonance imaging.

**Discussion:**

This protocol is the first adequately powered randomized clinical trial investigating the effects of augmenting ACLR with an LET in patients at high-risk of graft failure. The successful completion of this trial has the potential to change surgical practice and provide evidence for the role of the LET in ACLR.

**Trial registration:**

The trial is registered at ClinicalTrials.gov: NCT02018354, 23-12-2013.

## Background

The aim of anterior cruciate ligament reconstruction (ACLR) is to regain functional stability of the knee following anterior cruciate ligament (ACL) injury, allowing patients to return to their pre-injury level of activity. The reconstruction procedure has evolved significantly since the first ACLR was performed in the early 1900s [1] and while the techniques have changed, the goal of the surgery remains the same. A number of published meta-analyses have compared stability rates and functional outcomes of patellar tendon graft versus hamstring tendon graft and autograft versus allograft for ACL reconstruction [2–6]. The results have demonstrated that ACL reconstruction fails to restore normal knee stability [3, 5] regardless of graft choice, as rotational laxity [2, 4, 6] and anterior translation [2, 6] persist following surgery. Mohtadi et al. [7] conducted a level 1 randomized controlled trial comparing patella tendon (PT) grafts to single bundle hamstring (HT) and double bundle hamstring (DB) grafts. A significant number of patients in all three groups had rotational laxity characterized by a pivot shift greater or equal to 2 (HT – 19%; PT – 16%; DB – 21%). Abnormal tibial rotation has also been demonstrated in biomechanical assessment of traditional ACL reconstructions compared to the contralateral knee in functional tasks such as descending stairs [8], pivoting [8], and running [9]. Georgoulis et al. [10] later confirmed that a traditionally placed hamstring graft would not restore the normal knee joint kinematics. A positive pivot shift and ongoing rotational laxity has been shown to correlate with clinical outcome [11], which may be associated with graft failure and subsequent revision surgery. Studies investigating the epidemiology and risk factors for graft failure following an ACLR have found that younger patients (< 20 years old) [12–16], patients with physiologic knee hyperextension [17], and patients returning to pivoting sports [16] are at greater risk of re-injury.

Recently, a significant focus has been placed upon the anterolateral complex (ALC) of the knee, which includes the iliotibial band, anterolateral ligament (ALL) and lateral meniscus [18, 19]. Injuring these structures may contribute to rotational laxity about the knee [20, 21]. Cadaveric studies have demonstrated that when the both the ACL and ALL are sectioned, ACLR alone is insufficient to control knee rotation [22, 23]. Augmentation of ACLR with an extra-articular reconstruction has been recommended [22], and subsequently, the addition of a lateral extra-articular tenodesis (LET) was found to outperform an ALL reconstruction for controlling pivot shift [23].

Extra-articular reconstruction is not a new concept; early approaches to ACL deficiency included LET and several techniques were published [24]. Reports of poor results [25, 26] eventually resulted in this approach giving way to more advanced intra-articular procedures; however, a number of authors performed the extra-articular tenodesis along with intra-articular reconstruction and reported excellent results [27–30]. In 2015, Hewison et al. [31] conducted a meta-analysis of trials (*n* = 29; 8 randomized, 21 non-randomized) comparing ACLR alone to ACLR combined with an LET and found that the combined ACL and LET procedure significantly reduced rotational laxity assessed by pivot shift (odds ratio, 0.50 [95% confidence interval: 0.32 to 0.78], *p* = 0.002). There were no differences between groups for International Knee Documentation Scores (IKDC) or anterior laxity assessed by KT-1000/− 2000, though the majority of included studies demonstrated a high or unclear risk of bias and lacked sufficient sample size indicating a need for further research.

The goal of providing a rotationally stable knee following ACLR remains difficult to achieve. It is hypothesized that poor rotational control, and hence a positive pivot shift, may predispose patients to future graft failure and need for revision surgery. This is particularly problematic in young patients returning to pivoting sport, who have been shown to be at a much higher risk of early graft failure. A surgical procedure that addresses rotational control is therefore of utmost importance when treating the ACL deficient knee. The Stability Study is a pragmatic, multicenter, randomized clinical trial (RCT) where patients will be randomized to ACLR with or without an LET in a 1:1 ratio. The purpose of this study is to determine whether ACL reconstruction augmented with a modified Lemaire LET results in a reduced rate of graft failure compared to ACL reconstruction alone in patients who are at high risk of graft failure. To our knowledge, this will be the first level 1 study adequately powered to detect a clinically relevant reduction in graft failure, following a combination of intra-articular ACL reconstruction with lateral extra-articular tenodesis in patients who are deemed as being at higher risk of early graft failure. This study will answer important questions on how best to control rotational stability of the knee, and whether this has an effect on resultant graft failure in high risk individuals.

## Methods

### Study setting

**Fig. 1:**
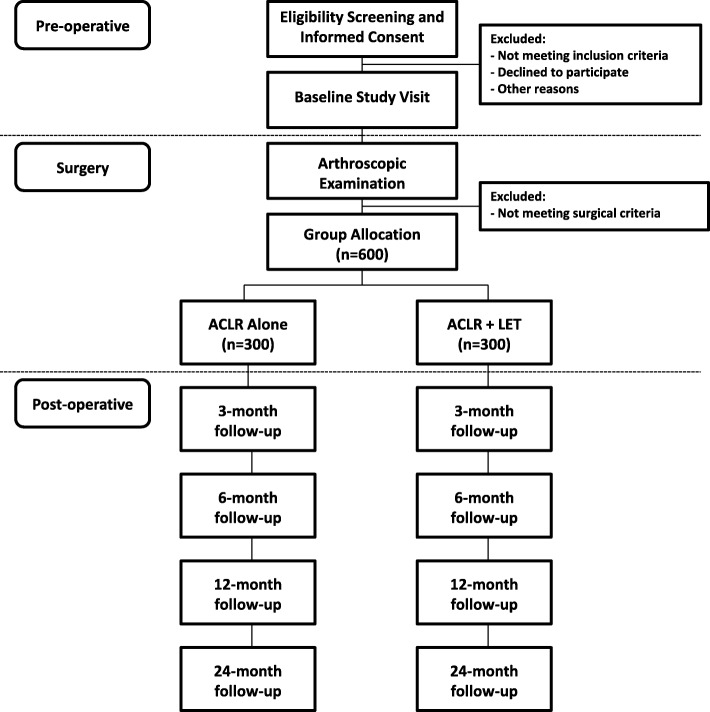
Study Flow Diagram

Patients will be recruited from orthopedic sport medicine clinics at 7 sites in Canada (*Fowler Kennedy Sport Medicine Clinic*, London ON; *McMaster University*, Hamilton ON; *Banff Sport Medicine Clinic*, Banff AB; *Pan Am Sport Medicine Clinic*, Winnipeg MB; *Fraser Health Authority*, New Westminster BC; *Queen’s University*, Kingston ON; *University of Calgary Sport Medicine Centre*, Calgary AB) and 2 sites in Europe (*Antwerp Orthopedic Center*, Antwerp BE; *University Hospitals Coventry and Warwickshire NHS Trust*, Coventry UK). Patients will be referred to orthopedic surgeons at these centres, where the clinician will screen for potential participants and provide information about the research study. See Fig. 1 for an outline of the study.

### Eligibility criteria

Patients will be eligible to participate if they have an ACL deficient knee and are skeletally mature (as defined by closed growth plates on plain radiograph) between 15 and 25 years of age. Patients must also have two or more of the following criteria: competitive pivoting sport, grade two (2) pivot shift or greater, generalized ligament laxity (Beighton score of four (4) or greater). Patients will not be eligible to participate if they have had previous ACLR on either knee, a multi-ligament injury (two or more ligaments requiring surgical attention), symptomatic articular cartilage defect requiring treatment other than debridement, varus or valgus malalignment greater than three (3) degrees, unable to speak or read English and unable to provide informed consent (Table 1).

**Table 1:** Eligibility Criteria

Inclusion Criteria	Exclusion Criteria
1. Skeletally mature between the ages of 15 and 25	1. Previous ACLR on either knee
2. ACL deficient knee	2. Multi-ligament injury (2+ ligaments requiring surgical attention)
3. Two or more of the following:	3. Symptomatic articular cartilage defect requiring treatment other than debridement
a) Competitive pivoting sport	4. Varus or valgus malalignment greater than 3 degrees
b) Pivot shift grade 2 or greater	5. Unable to speak or read English
c) Generalized ligamentous laxity (Beighton score of 4 or greater)	6. Unable to provide informed consent

### Intervention

All patients will undergo an anatomic ACLR. All ACLRs will be performed in a standardized fashion across sites. Specifically, surgeons will use a four-strand autologous hamstring autograft. If the diameter of the graft is found to be less than 8 mm, semitendinosus will be tripled (5 strand graft) providing a greater graft diameter. Femoral tunnels will be drilled using an anteromedial portal technique, with femoral fixation provided by an Endobutton or equivalent. Tibial fixation will be provided by interference screw.

#### Patients randomized to receive LET

All LETs will be performed in a standardized fashion across sites. Specifically, surgeons will make an oblique skin incision between the lateral epicondyle and Gerdy’s tubercle, measuring approximately 5 cm. A 1 cm wide × 8 cm long strip of iliotibial band is fashioned, leaving the Gerdy’s tubercle attachment intact. A No. 1 vicryl whip suture is applied to the free end, leaving the needle attached. The graft is then tunneled under the fibular collateral ligament (FCL) and attached to the femur with a Richards’ staple (Smith & Nephew), just distal to the intermuscular septum, proximal to the femoral insertion of the fibular collateral ligament. Fixation is performed with the knee at 70° flexion, neutral rotation. Minimal tension is applied to the graft. The free end is then looped back onto itself and sutured using the No. 1 vicryl.

All patients, regardless of group allocation will undergo identical postoperative rehabilitation. Focus is placed upon early range of motion and weight bearing as tolerated. No brace is used. Briefly, this includes:

- 0–6 weeks: General range of motion, swelling control, quadriceps activation, muscle stretching and strengthening.
- 6–12 weeks: Range of motion, muscle strength, proprioception, cardiovascular fitness.
- 3–6 months: Flexibility and sport specific muscle strengthening. Cardiovascular fitness.
- 6–9 months: Sport specific training.
- 9 months +: Return to sport if meeting functional requirements.

## Outcomes

### Primary outcome

The primary outcome is a composite endpoint of graft failure, defined as either: symptomatic instability requiring revision ACL surgery, or symptomatic instability with positive pivot shift or asymmetrical pivot shift greater than the contralateral side. The purpose of extra-articular reconstruction is to provide greater rotational control, and therefore potentially reduce the incidence of traumatic or atraumatic graft failure. Therefore, it follows that if a positive pivot shift is present, then the extra-articular reconstruction has failed.

### Secondary outcomes

Secondary outcome measures will include patient-reported quality of life and functional outcome measures, functional testing and biomechanical assessment, return to activity, imaging, cost effectiveness and adverse events.

#### Patient-reported quality of life

We will measure disease-specific quality of life using the *ACL Quality of Life Questionnaire* (ACL-QOL). The ACL-QOL has five domains that query physical symptoms, occupational concerns, recreational activities, lifestyle, and social and emotional aspects. Each item has one 100 mm visual analogue scale response option, with labeled anchors at 0 mm (e.g., extremely difficult) and 100 mm (e.g., not difficult at all). Scores are calculated by converting the average of each of the five domain scores to a total average score out of 100% where 100% represents the best possible score. The ACL-QOl has demonstrated validity [32] in patients with ACL injury and responsiveness to change [33] in patients following ACLR.

We will measure region-specific quality of life using the *Knee Osteoarthritis and outcomes Score* (KOOS). The KOOS is a 42-item knee-specific questionnaire with five separately reported domains, including pain (9 items), other symptoms (7 items), function in daily living (17 items), function in sports/recreation (5 items) and knee-related quality of life (4 items). Domain scores represent the average of all items in the domain standardized to a score from 0 to 100 (worst to best). This instrument has demonstrated reliability, validity and responsiveness in patients undergoing ACLR [34].

We will measure quality-adjusted life years (QALYs) using the *European Quality of Life Scale* (Euro-QoL) [35]. The EuroQoL comprises two sections, the *EQ-5D index* and the *EQ-5D visual analogue scale* (VAS). The EQ-5D index is a 5-item standardized generic measure of HRQOL that includes domains of mobility, self-care, usual activities, pain and discomfort and anxiety and depression. Each item is score using a 3-point response scale and each combination of response choices describes a health state (243 unique health states). Each health state can be converted to a utility value from 0 (worst) to 1.0 (best) using a scoring formula. The EQ-5D VAS is a 0 (worst) to 100 (best) scale that assesses patient-perceived health status. The EQ-5D index and VAS have demonstrated good test-retest reliability (0.73 and 0.70 respectively) and good cross-sectional construct validity in patients with rheumatoid arthritis [36] and those with osteoarthritis of the knee and is able to discriminate between functional classes in patients with arthritis.

#### Patient-reported functional outcomes

*The International Knee Documentation Committee Subjective Knee Form* (IKDC) is a knee-specific functional outcome that consists of 18-items asking patients about symptoms (7 items), sports activities (10 items) and function (1 item). Likert scales, a dichotomized item and 11-point rating scales make up the response options, and total score ranges from 0 (total limitation) and 100 (no limitations). The IKDC has shown adequate test-retest reliability and good construct validity in patients with issues at the knee [37].

*The Lower Extremity Functional Score* (LEFS) is a self-report functional outcome containing 20 items with five response options each. Responses range from 0 (extreme difficulty) to 4 (no difficulty) and the total score is found by summing the responses for a maximum score of 80, which indicates high functional level. The LEFS has demonstrated excellent test-retest reliability (0.94) and construct validity in patients with lower extremity issues [38].

*The 4-Item Pain Intensity Measure* (P4) is a four-item questionnaire that asks patients to report the amount of pain they experience throughout the day (morning, afternoon, evening) and with activity. Each item response is a visual analog scale ranging from 0 (no pain) to 10 (pain as bad as it can be). The total score is calculated by summing the four responses to a maximum score of 40. The P4 has demonstrated good test-retest reliability (0.78) and the ability to detect changes in pain intensity in patients with acute muscle and joint injuries [39].

#### Objective functional outcomes

We will assess *range of motion* (ROM) by measuring passive knee extension and active-assisted knee flexion. For passive knee extension, the patient is seated with both legs extended on a table, heel propped so that the calf and upper thigh clear the treatment table. We will instruct the patient to relax both quadriceps and hamstrings to assure passive measurement. For active-assisted knee flexion, the patient is seated with both legs extended on a table. We will instruct them to perform active-assisted knee flexion by placing one hand under their thigh to initiate flexion and then clasp both hands just below the tibial tuberosity. We will measure and record flexion and extension in degrees using a goniometer. For both measurements, we will centre the fulcrum of the goniometer over the lateral epicondyle of the femur. We will then align the stationary arm of the goniometer with the greater trochanter of the femur and the moving arm with the lateral malleolus at the ankle.

*Strength testing* will be performed using a computerized isokinetic dynamometer. Each test consists of six consecutive alternating knee flexion (three repetitions) and extension (three repetitions) movements and is assessed by using maximal concentric muscle actions at an angular velocity of 90°/s. During each test session, we will have the patient seated with his/her back against a backrest oriented at 80° above the horizontal and his/her hips in approximately 80° of flexion. We will secure the patient’s pelvis using a seatbelt oriented diagonally across the anterior superior iliac spines to the dynamometer seat and backrest. We will position the axis of rotation of the dynamometer lever arm coaxial to the lateral femoral epicondyle. Once we have the patient positioned correctly, we will familiarize them with the testing apparatus and ask them to perform at least four practice contractions before testing. During each test, we will provide the patient with a 30-s rest period between movements.

*The hop test* is a performance-based outcome measure designed to evaluate neuromuscular control, strength and confidence in the limb. The test is a combination of four different hop tests that incorporate a variety of movement principles (e.g., direction of change, speed, acceleration-deceleration) that mimic the demands of knee stability during sporting activities. We will ask patients to perform the single hop for distance test by standing on the leg to be tested, then hopping and landing on the same limb. The kinesiologist will measure and record the distance hopped at the level of the great toe. We will have patients complete the timed 6-m hop test by performing large one-legged hops in series over the total distance. The kinesiologist will start the stopwatch when the patient’s heel lifts from the starting position and will stop it at the moment the testing foot passes the finish line. We will have the patient complete the triple hops for distance test by standing on one leg, then performing three consecutive hops on the same leg landing as far as possible. We will measure and record the total distance covered by the three consecutive hops. We will ask patients to perform the crossover hop for distance by having the patient stand on the leg to be tested, then hop forward three times while alternately crossing over the width of the line (15-cm wide). We will measure and record the total distance covered by the crossover hops. We will offer a rest period between types of hop tests (up to two minutes) and between individual hop test trials if needed. Based on the performance of all four tests, we will calculate the limb symmetry index (test performance of the operative limb expressed as a percentage of the opposite limb), to differentiate and compare knee stability and rehabilitation strategies. This instrument has demonstrated validity and excellent test-retest reliability [40].

#### Biomechanical assessment

Drop vertical jump (DVJ) assessment will take place in the in the Wolf Orthopaedic Biomechanics Laboratory (WOBL) using motion analysis equipment. We will use autoreflective markers to track movements. We will instruct patientsto step off an elevated platform (box with a height of 31 cm) and land on a force plate with both feet. Upon contact with the force plate, we will have patients perform a vertical jump and re-land in the spot of initial contact. We will then use data collected by the force plates and reflective markers to calculate a peak knee abduction moment (simply defined as the distance between the joint axis and the vector produced by the ground reaction force). Peak knee abduction moment has been shown to predict the risk of ACL injury in young female athletes [41], and will therefore be used as a surrogate outcome for reinjury within our study.

During this task, we will have a clinician and researcher assess the biomechanics of the Drop Vertical Jump using a Clinician Rated Drop Vertical Jump Scale. This scale is being incorporated to test its validity and reliability. We will video record the patient’s torso and lower body while they perform this task. We will have the clinician and researcher review the video footage for comparison to their original scores.

#### Imaging

We will assess the articular cartilage in the lateral compartment of the knee, using quantitative *magnetic resonance imaging* (qMRI). Specifically, T1rho and T2 relaxation times are sensitive to changes in collagen and proteoglycan content of articular cartilage [42, 43], typically observed following ACL injury [44, 45]. Increases in T1rho or T2 relaxation time have been shown to be predictive of the development of OA [46]. Participants will undergo MRI at 24 months postoperative. Participants with a meniscal root tear will undergo MRI at 12 months and 24 months postoperative to assess healing. Scans will be performed on a 3 T Siemens Magnetom Trio magnet, and a 15-channel Siemens PRISMA knee coil (Siemens Medical Solutions, New Jersey, USA). qMRI pulse sequences consist of a Sagittal Multi-Echo Spin Echo T2 Mapping sequence, and a 16-shot Gradient Echo T1rho Mapping sequence.

We will also evaluate radiographical evidence of OA using plain anteroposterior, Rosenberg and lateral view radiographs. Joint space degeneration will be evaluated by a clinician using the Kellgren-Lawrence Grading Scale [47], which ranges from 0 (normal joint space) to grade 4 (large osteophytes, marked narrowing of joint space, severe sclerosis and deformity of bone contour).

#### Return to activity

The *MARX Activity Rating Scale* (MARS) is a four-item activity rating scale. The patient is asked to rate how often they were able to perform each activity (e.g. running, cutting, decelerating, and pivoting) in their most healthy and active state. The patient is provided with five categories of frequency of each functional activity, ranging from less than one time in a month to four or more times in a week. One point is allocated for each category of frequency and a maximum score of 16 points can be awarded. The MARS has demonstrated excellent test-retest reliability and construct validity in patients with disorders of the knee [48].

We will look at return to sport by asking patients to report their primary sport and level of participation prior to injury. Following surgery, we will ask patients to record the date they returned to their primary sport and level and provide reasons they chose not to return if so indicated.

#### Adverse events and cost outcomes

We will record *adverse events* including wound complications, infections, painful hardware, re-operation (e.g. meniscal injuries, etc), and radiographic evidence of osteoarthritis (radiographs are part of routine care).

A research assistant at each site will contact patients who have experienced an adverse event to capture cost data associated with the event. The cost form consists of 12 domains (emergency room visits and hospitalizations, family doctor visits, specialist visits and outpatient clinics, health care professional visits, tests, procedures and surgeries, prescription medications, supplies and equipment, over-the-counter medications, employment statues and time-off work from paid employment, assistance from others, assistance living and transportation and miscellaneous costs) that patients self-report.

### Participant timeline

After the patient has been screened and signed the informed consent document, we will schedule a pre-operative baseline appointment. At baseline, we will have patients undergo a clinical assessment, range of motion, complete the seven patient-reported questionnaires along with demographics and a pre-op return to sport questionnaire, and perform the strength testing. Post-operatively, we will see participants at 3 months, 6 months, 12 months and 24 months for further follow-up (Table 2).

**Table 2:** Participant Timeline

Assessments	Appointment					
	Baseline	Surgery	3 Months	6 Months	12 Months	24 Months
Demographics	X					
Surgery Forms		X				
ACLQOL	X		X	X	X	X
IKDC	X		X	X	X	X
KOOS	X		X	X	X	X
Euro-QOL	X		X	X	X	X
MARS	X		X	X	X	X
LEFS	X		X	X	X	X
P4	X		X	X	X	X
Return to Sport	X				X	X
ROM	X		X	X	X	X
Clinical Assessment	X		X	X	X	X
Radiographs	X				X	X
Adverse Event Form		X	X	X	X	X
Strength Testing	X			X	X	X
Hop Testing				X	X	X
Drop Vertical Jump Testing				X	X	
Root Tear MRI					X	X
Two Year MRI						X

### Recruitment

Patients presenting to the participating centres will first be diagnosed by an orthopaedic surgeon. After diagnosis, the surgeon will introduce and briefly outline the research study to the patient. The surgeon will then ask the patient if a member of the research team (graduate student or research assistant) can contact them with further information about the study. Upon receiving patient approval, a member of the research team will contact the patient with more information. The patient will be given the necessary time to consider participation in the study and will accept or decline once they have come to a decision.

Patients under the age of 18 will be provided with information about the study and will be asked to provide their consent along with legal guardian consent. Parents or guardians that are present at the appointment will be included in the informed consent process. If parents or guardians are not present the patient will be given the opportunity to discuss their participation with their parent or guardian before making a decision regarding trial participation.

### Randomization

#### Concealment mechanism/implementation

Potential participants will be screened by an independent clinician. Upon determining willingness to participate, confirming eligibility and meniscal status intra-operatively, a research assistant will enter the patient’s date of birth, sex and meniscus status before randomizing the patient via telephone or web-based randomization system to ACLR alone or ACLR with LET.

### Allocation (sequence generation)

All patients will be randomized by surgeon using a standard randomization design whereby each surgeon performs either ACLR alone or ACLR with LET in a 1:1 ratio. Randomization is stratified by surgeon, sex and meniscal tear status (presence or absence of meniscal repair requiring a change in postoperative rehabilitation) in permuted block sizes of two and four to ensure that the difference in outcome attributable to surgeon is equally dispersed between groups. Surgeons will be unaware of block order.

### Blinding

Surgeons, data collectors, and the data analyst will be blind to group allocation. The addition of a LET procedure results in a unique incision on the lateral side of the knee between the lateral epicondyle and Gerdy’s tubercle. The unblinded member of the research team will place an opaque adhesive across the lateral side of the knee covering the area where a LET incision could be seen for clinical assessment and range of motion. Clinical assessment will be completed by an independent assessor (an experienced surgeon that did not perform the surgical procedure on the patient). Participants will be asked to wear a tubigrip sleeve over the operative knee during all functional testing to conceal the incisions.

### Sample size

We estimate that within this group of high-risk patients that the absolute risk (AR) of graft failure (as defined above) in the ACLR group will range from 25 to 35%. We would consider a relative reduction in re-rupture rate of at least 40% to merit a change in practice (i.e. of sufficient magnitude to warrant the additional costs related to the procedure). Thus, with 255 patients per group and a type 1 error rate of 5% we would have approximately 80% power to detect a relative risk reduction (RRR) in rate of re-rupture in the LET of 40% or greater. Because we expect a combined withdrawal and lost-to-follow-up rate of about 15%, we will recruit a total of 600 patients (300 per group).

### Plan for statistical analysis

We will determine the absolute risk of graft failure in each group, calculate a relative risk (RR) and risk difference (RD) of graft failure with 95% confidence intervals around the estimate and use a Mantel Haentzel Test (random effect of surgeon) to determine the significance of the association between the addition of LET and graft failure rates. We will calculate the number needed to treat (NNT) to describe the number of patients who need to receive LET to prevent one graft failure over the first two postoperative years. We will calculate the mean and standard error (ACL-QOL, KOOS, EuroQoL) for each group at each time point and calculate the mean between-group difference with 95% confidence interval at 1 and 2 years postoperative. We will perform mixed model analyses, where time is a categorical fixed effect; treatment group, and sex are fixed effects, and patient and surgeon are random effects, to determine whether the difference between groups is of sufficient magnitude to make a definitive conclusion about the effectiveness of ACLR with LET compared to ACLR alone. Then, we will plot each health status score by time and by group to illustrate the change over time. We will conduct a similar analysis for all continuous outcome variables. Finally, for ease of interpretability, we will calculate the number of patients in each group who achieve a change at least as large as the MCID for an individual patient. We will present the NNT to describe the number of patients who need to undergo LET for one person to achieve an important change in HRQOL.

For qMRI analysis, T1rho and T2 relaxation maps are generated using software developed in-house by fitting image intensities of the T1rho and T2 weighted images pixel-by-pixel to the eq. S(TE) ∝ exp.(−TE/T2) using a Levenberg-Marquardt mono-exponential fitting algorithm. Load-bearing central, anterior, and posterior regions of medial and lateral articular cartilage of the femur and tibia are manually segmented and analyzed using 3D Slicer software. The reader is blind to scan order. We will use an independent t-test to evaluate whether the T1rho and T2 relaxation times between groups for all nine cartilage segments are statistically significant. We will present means, standard deviations and 95% confidence intervals for interpretation.

## Data collection methods

The timing of follow-up assessments corresponds with regular visits to the surgeon following ACLR. During these visits, the research assistant will administer the questionnaires to ensure they are complete. Alternatively, the patient can complete the questionnaires online within the visit window by directly accessing the electronic data capture (EDC) system (*Empower Health Research*). A blinded surgeon will complete the knee exam and a blinded kinesiologist will conduct the hop and strength tests within the clinic at 6, 12, and 24 months postoperative. Participants will complete the DVJ test at their 6- and 12-month visit in WOBL. MRI’s will be conducted at Robarts Research Institute located adjacent to the lead site and a radiologist, blind to group allocation, will report MRI findings using a standardized form.

## Discussion

ACL injury is one of the most common orthopedic knee injuries and, despite advances in surgical technique and rehabilitation protocols, up to 30% of patients will re-tear their graft or suffer a contra-lateral ACL injury with younger age groups particularly at risk [16, 49]. Persistent instability leads to worse patient outcomes following ACLR and biomechanical cadaveric studies have shown that an ACLR alone may be insufficient to restore normal rotation. A 2015 meta-analysis of 29 studies (8 randomized, 21 non-randomized) comparing ACLR alone with ACLR and LET found the addition of a LET reduces rotational laxity by a statistically significant amount however, studies comparing the two procedures demonstrated limitations including small sample size and potential risk of bias [31].

The protocol for the *Stability Study* is the first adequately powered RCT to study the effects of whether augmenting ACLR with a LET reduces failure in young, high-risk patients. Two further potential strengths of this study are the addition of functional and biomechanical testing and quantitative MRI to the protocol. Functional and biomechanical testing are measures of neuromuscular training and patterning that require muscle strength, proprioception and balance to complete [50], all of which are important components in rehabilitation and injury prevention [51, 52]. Despite high re-injury rates and issues returning to pre-injury levels of sport following ACLR, few high-quality studies include comparison of functional and biomechanical outcomes between groups [53].

Concerns that the addition of an LET to the lateral side of the joint may increase the risk of post-traumatic OA will be addressed by the addition of qMRI to the trial. A recent systematic review found that radiographic evidence of OA was not significantly increased in patients where ACLR was augmented with LET compared to ACL alone [54]. Patients were assessed for radiographic evidence of OA however follow-up length and results were variable between studies. Using qMRI to evaluate cartilage degeneration allows us to compare articular cartilage health between groups through T1Rho and T2 relaxation times; increased relaxation times have been shown to be predictive of the development of OA [46].

High graft failure rates following ACLR, particularly in young, active patients, are concerning and need to be addressed. It has been suggested that poor rotational control following ACLR may predispose patients to graft failure, which leads us to believe there may be a role for augmenting the ACLR with an extra-articular reconstruction. Previous studies combining ACLR and a LET showed promising results, though small sample sizes and potential biases limit the conclusions we can make. The Stability study protocol is for a multicenter, international, adequately powered RCT comparing ACLR alone to ACLR combined with an LET. This study can provide level-one evidence regarding the effect augmenting an ACLR with a LET has on graft stability, patient quality of life, functional performance, biomechanics, and radiographic outcomes, and has the potential to change clinical practice.

## Abbreviations

ACL: Anterior cruciate ligament
ACLQOL: Anterior Cruciate Ligament Quality of Life Questionnaire
ACLR: Anterior cruciate ligament reconstruction
AE: Adverse event
AR: Absolute risk
DVJ: Drop Vertical Jump
EDC: Electronic data capture
Euro-Qol: European Quality of Life Scale
HRQOL: Health-related quality of life
IKDC: International Knee Documentation Committee Subjective Knee Form
KOOS: Knee Osteoarthritis and Outcome Scores
LEFS: Lower Extremity Functional Score
LET: Lateral extra-articular tenodesis
MARS: Marx Activity Rating Scale
MRI: Magnetic resonance imaging
NNT: Number needed to treat
OA: Osteoarthritis
P4: Four-Item Pain Intensity Measure
QALY: Quality-adjusted life years
qMRI: Quantitative magnetic resonance imaging
RCT: Randomized clinical trial
RD: Risk difference
ROM: Range of motion
RR: Relative risk
RRR: Relative risk reduction
VAS: Visual analog scale
WOBL: Wolf Orthopaedic Biomechanics Laboratory
